# Induced pluripotent stem cell technology as diagnostic tool in patients with suspected ornithine transcarbamylase deficiency lacking genetic confirmation

**DOI:** 10.1016/j.ymgmr.2023.101007

**Published:** 2023-09-06

**Authors:** Adhuresa Ramosaj, Palak Singhal, André Schaller, Alexander Laemmle

**Affiliations:** a Division of Pediatric Endocrinology, Diabetology and Metabolism, Department of Pediatrics, Inselspital, Bern University Hospital, University of Bern, Bern, Switzerland; b University Institute of Clinical Chemistry, Inselspital, Bern University Hospital, University of Bern, Bern, Switzerland; c Department of Human Genetics, Inselspital, Bern University Hospital, Department for BioMedical Research (DBMR), University of Bern, Bern, Switzerland

**Keywords:** Urea cycle disorder, Ornithine transcarbamylase deficiency, Human induced pluripotent stem cell technology, hiPSC-derived hepatocytes, X-linked disease, X chromosomal inactivation

## Abstract

Ornithine transcarbamylase (OTC) deficiency (OTCD) is an X-linked urea cycle disorder. In females - undergoing random X chromosomal inactivation (XCI) - disease severity depends on the XCI pattern. Hence, female OTCD subjects with favorable XCI display normal OTC expression and activity and are healthy carriers. Whereas females undergoing less favorable XCI may suffer from severe and fatal OTCD. In approximately 20% of patients with biochemical evidence of OTCD, no mutation can be identified hampering definitive diagnosis and adequate treatment.

Here, we describe a female patient with high suspicion of OTCD in whom molecular genetic work-up did not reveal pathogenic variants in the *OTC* gene. In her case, this was particularly challenging, since she was awaiting liver transplantation due to metabolic instability. In order to substantiate the suspected diagnosis of OTCD, we applied our previously reported in vitro OTCD liver disease model. Patient-derived skin fibroblasts were reprogrammed into human induced pluripotent stem cells (hiPSCs) followed by differentiation into hepatocytes (hiPSC-Heps). Among five randomly selected hiPSC clones - differentiated into hiPSC-Heps - one clone expressed OTC protein, while the four remaining clones lacked OTC expression, supporting the patient's suspected diagnosis of OTCD.

To conclude, we demonstrate that hiPSC technology is a powerful diagnostic tool to substantiate the suspected diagnosis of OTCD in patients lacking genetic confirmation. Furthermore, selecting clones that exclusively express the wild-type OTC protein, could be used strategically as cellular therapy in future. Ultimately, this approach might be applicable to virtually any X-linked disease.

**Synopsis:**

Induced pluripotent stem cell technology is a powerful diagnostic tool to substantiate the suspected diagnosis of OTCD in patients lacking genetic confirmation.

## 1 Introduction

Ornithine transcarbamylase (OTC; EC 2.1.3.3) deficiency (OTCD; OMIM #311250) is a potentially life-threatening X-linked urea cycle disorder (UCD) [[1], [2], [3]]. OTC and the other urea cycle enzymes (UCEs), e.g. carbamoylphosphate synthetase 1 (CPS1; EC 6.3.4.16), are expressed in hepatocytes and detoxify ammonia – the waste product from protein digestion - into non-toxic urea in several consecutive enzymatic reactions. UCDs are caused by genetic defects in one of the UCEs and are accompanied by recurrent metabolic crisis with hyperammonemia [4].

Suspicion of OTCD is generally raised due to elevated plasma ammonia levels and additional biochemical laboratory parameters such as specific plasma amino acid changes like increased glutamine and decreased citrulline as well as increased urinary orotic acid [2]. Suspicion of OTCD requires rapid disease confirmation either by assessing OTC enzyme activity or by molecular genetic analysis [5,6]. While determination of hepatic OTC enzyme activity requires a liver biopsy which is often not a feasible option due to its invasive character, molecular genetic analysis of the *OTC* gene is the first line diagnostic approach [2].

However, in about 20% of patients with distinct biochemical evidence of OTCD, no pathogenic variants are found in the *OTC* gene hampering definitive diagnosis and adequate treatment [7].

Current treatment options are limited and consist of dietary protein restriction and supplementation of amino acid mixtures and nitrogen scavengers [2]. Liver transplantation is curative, however, this approach is comparatively invasive and often limited due to liver donor shortage [8].

In females - undergoing random X chromosomal inactivation (XCI) - disease severity largely depends on the XCI pattern of the *OTC* gene [[9], [10], [11], [12]]. Hence, female OTCD subjects with favorable XCI will have near to normal OTC expression and are considered healthy carriers. Whereas, females undergoing less favorable XCI may suffer from severe and fatal OTCD [13].

Here, we describe a female patient in whom molecular genetic work-up did not confirm the suspected diagnosis of OTCD. In her case, this was particularly challenging since she was awaiting liver transplantation due to recurrent metabolic decompensations. In order to substantiate the suspected diagnosis of OTCD, we applied our previously reported in vitro OTCD liver disease model of reprogramming patient-derived skin fibroblasts into human induced pluripotent stem cells (hiPSCs) followed by consecutive differentiation into hepatocytes (hiPSC-Heps) and characterization of OTC expression [14].

## 2 Case report

We describe a 17-year old female patient highly suspicious of OTCD. In her first year of life, she suffered from a metabolic decompensation with hyperammonemia (maximal level measured approximately 700 μM; reference <50 μM). While her laboratory work up including plasma ammonia levels and amino acid profiles with elevated glutamine and low citrulline levels as well as elevated urinary orotic acid excretion were highly suggestive of OTCD, her family history was unremarkable regarding OTCD. Due to repeatedly low plasma citrulline levels and absence of elevated plasma and urinary argininosuccinate and homocitrulline, potential differential diagnosis such as argininosuccinate synthetase (ASS) deficiency (ASSD; OMIM #215700), argininosuccinate lyase (ASL) deficiency (ASLD; #207900) and hyperornithinemia-hyperammonemia-homocitrullinuria (HHH syndrome; OMIM #238970) were ruled out. Molecular genetic testing using Whole Exome Sequencing did not reveal pathogenic variants in the *OTC* gene or in any of the other UCEs or transporters for citrin (*SLC25A13*) and ornithine (*SLC25A15*). Hence, as observed in about 20% of patients with biochemical evidence of OTCD, molecular genetic testing lacked confirmation of diagnosis. Therapy for OTCD was immediately installed and consisting of a protein-restricted diet and substitution with amino acid mixture, sodium benzoate, phenylbutyrate, citrulline and arginine. Despite adherence to therapy, over the following years the patient recurrently suffered from metabolic crisis necessitating prompt and frequent emergency hospitalizations. Laboratory investigations - specifically plasma ammonia levels - between these episodes were normal. Remarkably, from a clinical point of view the patient had a completely normal neurodevelopment and neurocognition. However, her quality of life has been severely impaired since early childhood not only due to recurrent hospitalizations but also due to constant fear of life-threatening metabolic decompensations. Therefore, the patient has been listed for liver transplantation despite lack of molecular genetic confirmation of OTCD.

To substantiate the suspected diagnosis of OTCD we used the hiPSC technology.

## 3 Material and methods

### 3.1 Molecular genetic analysis

Molecular genetic testing was conducted on patient's DNA extracted from fibroblasts using Whole Exome Sequencing (TWIST Biosciences) including CNV analysis. Several genes encoding UCEs (CPS1, OTC, ASS, ASL and ARG1), *N*-acetylglutamate synthase (NAGS) and transporters for citrin (*SLC25A13*) and ornithine (*SLC25A15*) were analyzed.

### 3.2 Fibroblast reprogramming into hiPSCs and directed differentiation into hiPSC-Heps

Dermal skin fibroblasts from our patient (designated Subject OTCD_4) were obtained by skin punch biopsy. Three culture flasks of patient's skin fibroblasts (fibroblast culture #1, #2 and #3) at passage number three were independently reprogrammed into hiPSCs by episomal reprogramming as described previously [15]. Of over 50 hiPSC clones generated in parallel, five randomly selected hiPSC clones - clones G, T, K, N and S - were passaged seven times prior to directed differentiation into hiPSC-Heps according to our previously published protocol [14,16]. HiPSC clones G and T were derived from reprogramming fibroblast culture #1; hiPSC clones K, N from reprogramming fibroblast culture #2; and hiPSC clone S from reprogramming fibroblast culture #3. At day 22 of hepatocyte differentiation, hiPSC-Heps derived from OTCD_4 hiPSC clones G, T, K, N and S as well as from a previously established healthy control hiPSC line (Ctrl_1) with a passage number of 49 passages [16] were harvested and characterized for OTC and CPS1 expression by Western blot analysis as previously described [14].

### 3.3 Estimated costs and time frame to generate and characterize patient-derived hiPSC-Heps

The costs for laboratory consumables required to reprogram patient-derived fibroblasts into hiPSCs and directed differentiation into hiPSC-Heps and consecutive characterization depend largely on whether there is already an infrastructure available for hiPSC technology. The estimated costs for this project in our laboratory with already available infrastructure are at least 3000 US $. Not included in these calculations are the costs for working hours. The required time to generate and characterize patient-derived hiPSC-Heps from skin fibroblasts gained from a skin punch biopsy is at least three months.

## 4 Results and discussion

**Fig. 1:**
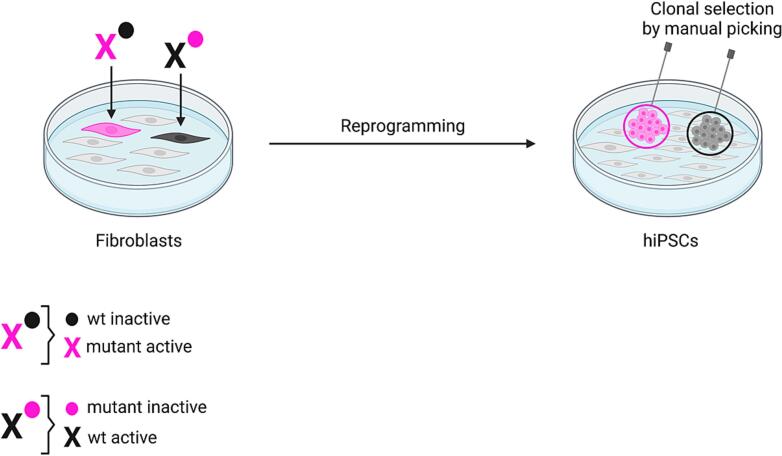
Scheme representing clonal selection of hiPSCs upon reprogramming patient-derived skin fibroblasts. OTCD_4 patient-derived dermal skin fibroblasts were reprogrammed into hiPSCs. The following mechanism of X chromosomal inactivation is proposed. Each fibroblast has inactivated one of both X chromosomes (circle for inactive X chromosome; X for active X chromosome) and either expresses the mutant (pink) or the wild-type (wt, black) *OTC* allele. Upon reprogramming a specific fibroblast into a specific clone of hiPSCs the X inactivation status is conserved within that clone either expressing mutant or wild-type *OTC* allele. Figure created with BioRender.com.

**Fig. 2:**
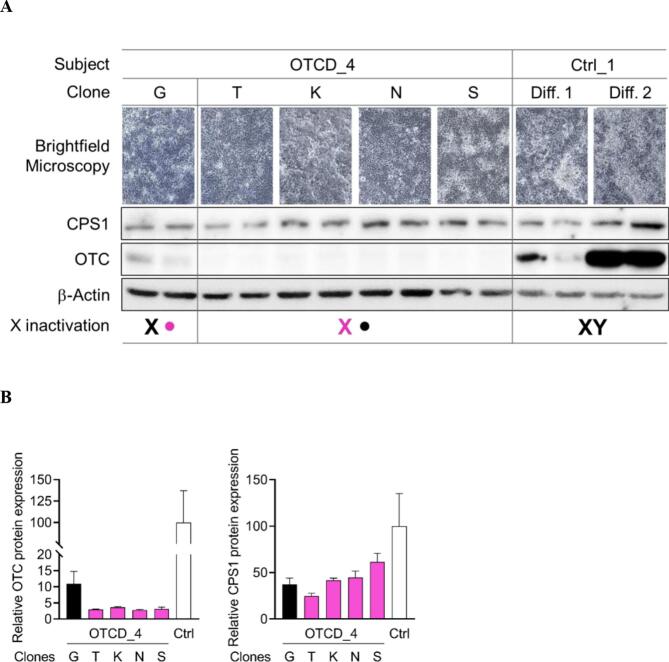
OTC protein expression in selected hiPSC clones differentiated into hiPSC-Heps. (A) Randomly selected hiPSC clones G, T, K, N and S from OTCD_4 patient and hiPSCs from a healthy control subject (Ctrl_1) were differentiated into hiPSC-Heps. Prior to cell harvest after 22 days of differentiation brightfield microscopy images were taken revealing hepatocyte-specific morphology. Harvested hiPSC-Heps were analyzed for OTC and CPS1 expression by Western blot. B-Actin served as loading control. The here proposed X inactivation status of the OTCD_4 hiPSC clones is based on the OTC expression. Clone G expresses wild-type (black X) *OTC* allele, clones T, K, N and S express mutant (pink X) *OTC* allele, and Ctrl_1 is a hemizygous male proband expressing wild-type *OTC* allele. (B) Relative OTC and CPS1 protein expression in OTCD_4 compared to Ctrl_1 hiPSC-Heps.

Dermal skin fibroblasts from OTCD_4 patient were reprogrammed into hiPSCs and over 50 different clones were generated and manually selected (Fig. 1). Based on our previous observation in hiPSCs derived from a female patient (OTCD_2) with genetically confirmed OTCD [14], we hypothesized that among the here generated OTCD_4 hiPSC clones some would exclusively express wild-type *OTC* allele, while others would exclusively express mutant *OTC* allele (Fig. 1). Therefore, we randomly selected five hiPSC clones G, T, K, N and S generated by reprogramming skin fibroblasts from OTCD_4 patient (Fig. 1) and a control hiPSC line (Ctrl_1) and differentiated them into hiPSC-Heps according to our previously established protocol [14]. At the end of the differentiation process after 22 days, hiPSC-Heps revealed hepatocyte-like morphology as assessed by brightfield microscopy (Fig. 2A). While hiPSC-Heps derived from clone G expressed OTC protein, hiPSC-Heps derived from clones T, K, N and S revealed (virtually) absent OTC protein expression as assessed and quantified by Western blot (Fig. 2A and B). These results suggest different X chromosomal inactivation status (X inactivation) of the here investigated clones (Fig. 2A). Presumably, clone G inactivated the X chromosome harboring the mutant *OTC* allele thus expressing wild-type OTC protein (Fig. 2A). Whereas clones T, K, N and S inactivated the X chromosome harboring the wild-type *OTC* allele thus expressing mutated OTC protein which could not be detected by Western blot (Fig. 2A). Consistent results were obtained when determining the OTC activity and ureagenesis, revealing clearly higher levels in hiPSC-Heps derived from clone G compared to the other OTCD_4 patient-derived clones (data not shown since assays revealed results close to the detection limits). Of note, when comparing Western blot results from the OTCD_4 patient with the results received from the control subject (Ctrl_1), OTC protein expression is clearly lower in all patient-derived clones including clone G (Fig. 2A and B). This could be attributed to the fact that the Ctrl_1 hiPSC line was established previously, had a distinct higher passage number and thus was potentially able to differentiate into more mature hiPSC-Heps. Thus, OTC expression should primarily be compared among the hiPSC-Heps derived from different clones from OTCD_4 patient generated simultaneously and not with Ctrl_1 hiPSC-Heps. Further, we cannot exclude that X chromosomal inactivation might not be complete, i.e. individual clones might have different degrees of both alleles active or a mixed population as recently reviewed [17]. To better characterize our cells, we analyzed CPS1, the rate-limiting UCE, which is a hepatocyte-specific marker [14]. CPS1 was robustly expressed in all our hiPSC-Heps (Fig. 2A and B) as were several other UCEs (data not shown) demonstrating that we generated mature hepatocytes from all OTCD_4 hiPSC clones and Ctrl_1 hiPSC line. Like for OTC protein, CPS1 protein expression was highest in Ctrl_1 hiPSC-Heps. However, the difference between CPS1 expression in Ctrl_1 versus OTCD_4 hiPSC-Heps was far less pronounced than for OTC protein expression (Fig. 2A and B) in line with the suspected diagnosis of OTCD.

Taken together, these results demonstrate that in four out of five investigated OTCD_4 hiPSC clones OTC expression was absent while CPS1 expression was robust in all hiPSC-Heps supporting and substantiating the patient's suspected diagnosis of OTCD.

## 5 Conclusion

To conclude, we demonstrate that hiPSC technology is a powerful diagnostic tool to substantiate the suspected diagnosis of OTCD in patients in whom molecular genetic testing is not conclusive. We suggest implementing this diagnostic tool in specific clinical situations, particularly in patients with suspicion of OTCD awaiting liver transplantation. We recommend performing this diagnostic procedure in a routine or research laboratory specialized in hiPSC technology and with experience in OTCD.

Furthermore, our approach of clonal selection of reprogrammed cells could be applied therapeutically by selecting clones exclusively expressing the wild-type (non-mutated) OTC protein. Ultimately, this approach might be applicable to virtually any X-linked disease.

## Author contributions

AL designed the project, generated the hiPSCs and differentiated them into hiPSC-Heps and performed all experiments using hiPSC-Heps and wrote the manuscript. AR isolated genomic DNA and RNA and contributed to making the figs. AS performed genetic analysis and assisted with the transcript analysis. PS performed all the RNA experiments and was involved in the mutation analysis.

## Funding

AL was funded by “Batzenbär-Stiftung des Inselspitals”.

## Informed consent and ethical approval

Written informed consent was obtained from the patient. This study was approved by the local ethics committee in Bern, Switzerland (project ID: 2020-02979).

## Declaration of Competing Interest

All authors declare no conflict of interest.

## Data Availability

Data will be made available on request.

## References

[1] Kolker S., Garcia-Cazorla A., Valayannopoulos V., Lund A.M., Burlina A.B., Sykut-Cegielska J. (2015). The phenotypic spectrum of organic acidurias and urea cycle disorders. Part 1: the initial presentation. J. Inherit. Metab. Dis..

[2] Haberle J., Burlina A., Chakrapani A., Dixon M., Karall D., Lindner M. (2019). Suggested guidelines for the diagnosis and management of urea cycle disorders: first revision. J. Inherit. Metab. Dis..

[3] Caldovic L., Abdikarim I., Narain S., Tuchman M., Morizono H. (2015). Genotype-phenotype correlations in ornithine Transcarbamylase deficiency: a mutation update. J. Genet. Genom..

[4] Matsumoto S., Haberle J., Kido J., Mitsubuchi H., Endo F., Nakamura K. (2019). Urea cycle disorders-update. J. Hum. Genet..

[5] Rapp B., Haberle J., Linnebank M., Wermuth B., Marquardt T., Harms E. (2001). Genetic analysis of carbamoylphosphate synthetase I and ornithine transcarbamylase deficiency using fibroblasts. Eur. J. Pediatr..

[6] Engel K., Nuoffer J.M., Muhlhausen C., Klaus V., Largiader C.R., Tsiakas K. (2008). Analysis of mRNA transcripts improves the success rate of molecular genetic testing in OTC deficiency. Mol. Genet. Metab..

[7] Yamaguchi S., Brailey L.L., Morizono H., Bale A.E., Tuchman M. (2006). Mutations and polymorphisms in the human ornithine transcarbamylase (OTC) gene. Hum. Mutat..

[8] Yu L., Rayhill S.C., Hsu E.K., Landis C.S. (2015). Liver transplantation for urea cycle disorders: analysis of the united network for organ sharing database. Transplant. Proc..

[9] Batshaw M.L., Tuchman M., Summar M., Seminara J. (2014). Members of the urea cycle disorders C. A longitudinal study of urea cycle disorders. Mol. Genet. Metab..

[10] Maestri N.E., Lord C., Glynn M., Bale A., Brusilow S.W. (1998). The phenotype of ostensibly healthy women who are carriers for ornithine transcarbamylase deficiency. Medicine..

[11] McCullough B.A., Yudkoff M., Batshaw M.L., Wilson J.M., Raper S.E., Tuchman M. (2000). Genotype spectrum of ornithine transcarbamylase deficiency: correlation with the clinical and biochemical phenotype. Am. J. Med. Genet..

[12] Maestri N.E., Brusilow S.W., Clissold D.B., Bassett S.S. (1996). Long-term treatment of girls with ornithine transcarbamylase deficiency. N. Engl. J. Med..

[13] Gyato K., Wray J., Huang Z.J., Yudkoff M., Batshaw M.L. (2004). Metabolic and neuropsychological phenotype in women heterozygous for ornithine transcarbamylase deficiency. Ann. Neurol..

[14] Laemmle A., Poms M., Hsu B., Borsuk M., Rufenacht V., Robinson J. (2022). Aquaporin 9 induction in human iPSC-derived hepatocytes facilitates modeling of ornithine transcarbamylase deficiency. Hepatology.

[15] Okita K., Matsumura Y., Sato Y., Okada A., Morizane A., Okamoto S. (2011). A more efficient method to generate integration-free human iPS cells. Nat. Methods.

[16] Lee-Montiel F.T., Laemmle A., Charwat V., Dumont L., Lee C.S., Huebsch N. (2021). Integrated isogenic human induced pluripotent stem cell-based liver and heart microphysiological systems predict unsafe drug-drug interaction. Front. Pharmacol..

[17] Dandulakis M.G., Meganathan K., Kroll K.L., Bonni A., Constantino J.N. (2016). Complexities of X chromosome inactivation status in female human induced pluripotent stem cells-a brief review and scientific update for autism research. J. Neurodev. Disord..

